# Exploring the Mechanism of Hawthorn Leaves Against Coronary Heart Disease Using Network Pharmacology and Molecular Docking

**DOI:** 10.3389/fcvm.2022.804801

**Published:** 2022-06-16

**Authors:** Jie Ding, Jun Wu, Haoran Wei, Sui Li, Man Huang, Yan Wang, Qin Fang

**Affiliations:** ^1^Division of Cardiology, Department of Internal Medicine, Tongji Hospital, Tongji Medical College, Huazhong University of Science and Technology, Wuhan, China; ^2^Hubei Key Laboratory of Genetics and Molecular Mechanisms of Cardiological Disorders, Huazhong University of Science and Technology, Wuhan, China; ^3^Department of Gastroenterology, Hubei No. 3 People’s Hospital of Jianghan University, Wuhan, China

**Keywords:** hawthorn leaves, coronary heart disease, network pharmacology, molecular docking, inflammation

## Abstract

Hawthorn leaves, which is a traditional Chinese medicine (TCM), has been used for treating coronary heart disease (CHD) for a long time in China. But the limited understanding of the main active components and molecular mechanisms of this traditional medicine has restricted its application and further research. The active compounds of hawthorn leaves were obtained from TCMSP database and SymMap database. The targets of it were predicted based on TCMSP, PubChem, Swiss Target Prediction, and SymMap database. The putative targets of CHD were gathered from multi-sources databases including the Online Mendelian Inheritance in Man (OMIM) database, the DrugBank database, the GeneCards database and the DisGeNet database. Network topology analysis, GO and KEGG pathway enrichment analyses were performed to select the key targets and pathways. Molecular docking was performed to demonstrate the binding capacity of the key compounds to the predicted targets. Furthermore, RAW264.7 cells stimulated by lipopolysaccharides (LPS) were treated with three effective compounds of hawthorn leaves to assess reliability of prediction. Quercetin, isorhamnetin and kaempferol were main active compounds in hawthorn leaves. Forty four candidate therapeutic targets were identified to be involved in protection of hawthorn leaves against CHD. Additionally, the effective compounds of it had good binding affinities to PTGS2, EGFR, and MMP2. Enrichment analyses suggested that immune inflammation related biological processes and pathways were possibly the potential mechanism. Besides, we found that three predicted effective compounds of hawthorn leaves decreased protein expression of PTGS2, MMP2, MMP9, IL6, IL1B, TNFα and inhibited activation of macrophage. In summary, the present study demonstrates that quercetin, kaempferol and isorhamnetin are proved to be the main effective compounds of hawthorn leaves in treatment of CHD, possibly by suppressing expression of PTGS2, MMP2, MMP9, inflammatory cytokines and macrophages viability. This study provides a new understanding of the active components and mechanisms of hawthorn leaves treating CHD from the perspective of network pharmacology.

## Introduction

Coronary heart disease (CHD), one of the leading causes of human death in the world, is characterized by atherosclerosis of the coronary arteries, narrowing or occluding the vascular lumen, leading to myocardial ischemia, hypoxia or necrosis (1). Coronary artery stenosis or plaque rupture often causes myocardial ischemia or infarction, which can lead to heart failure even death (2). Drug therapy, one of the most important methods to treat CHD, includes nitrates drugs, β-receptor blockers, antithrombotic drugs, calcium channel blockers, renin-angiotensin system inhibitors and lipid-lowering drugs, which are usually aimed to act on individual targets (3). However, the effects of these drugs can’t eliminate vascular stenosis, especially, long-term use of some drugs may lead to side effects or drug resistance (4, 5). So, drugs with multiple active ingredients and multiple targets are urgently needed.

Traditional Chinese Medicine (TCM) has been used for ages to treat a variety of diseases including CHD and its related diseases. The diversity of components and mechanisms of action is an important feature of TCM (6). Hawthorn (Also known as Crataegus pinnatifida Bge) belongs to the genus crataegus of the rosaceae family. It is used as a source of food and medicine, and widely distributed in the northeast part of China (7). Currently, many researchers have put their focus on hawthorn not only for its fruit but also for its leaves. Hawthorn leaves, as raw materials of herbal medicines, have been proven to have a variety of pharmacological properties, including preventing or treating cardiovascular diseases, improving circulation of coronary, lowering blood lipids and preventing hypertension (8). Besides, adjusting digestive function disorder is also one of its important effects (9). A number of domestic and foreign studies have shown that hawthorn leaves have therapeutic effects on CHD (7, 10). Compared with single-target drugs, although hawthorn leaves have a richer array of active components and potential mechanisms, the details about bioactive compounds and mechanisms of action are rarely reported.

Network pharmacology is an important tool to explore the active components and action mechanism of TCM. It analyzes the mechanisms of action of drugs from a systematic and comprehensive perspective (11). So far, many studies exploring the active components and mechanisms of TCM in the prevention or treatment of CHD through network pharmacology have obtained satisfactory results (12–14). Approaches based on network pharmacology are promising research tools that can help us understand the relationship between drugs and disease in a more comprehensive and detailed way (15, 16).

In this study, approaches based on network pharmacology were used to investigate underlying mechanism of action of hawthorn leaves in treatment of CHD. The purpose of this study was to explore main active components and possible mechanisms of hawthorn leaves in the treatment of CHD from the perspective of network biology.

## Materials and Methods

### Identification of Active Ingredients

The Traditional Chinese Medicine Systems Pharmacology Database (TCMSP)^1^ (17) and SymMap Databases^2^ were used to identify bioactive ingredients of hawthorn leaves. Pharmacokinetic parameters of each compound [absorption, distribution, metabolism, and excretion (ADME)] in hawthorn leaves were determined. All compounds with oral bioavailability (OB) ≥ 30 % and drug-likeness (DL) ≥ 0.18 were retrieved for subsequent research. OB is a pharmacokinetic parameter, representing the relative amount of a drug absorbed into the systemic circulation after oral administration. DL is a qualitative characteristic used to evaluate the possibility of converting a compound into a drug.

### Prediction of Putative Targets of Hawthorn Leaves

The supposed targets of effective compounds were picked up from the TCMSP database (17; see text footnote 1), the PubChem database (18),^3^ the Swiss Target Prediction database (19)^4^ and the SymMap Databases (see text footnote 2). Then, uniprot sites^5^ were used to process all the names of targets and normalize the gene information following the standards of human origin. Detailed information of supposed targets is showed in Supplementary Table 1.

### Identification of Coronary Heart Disease Related Targets

Related CHD targets were collected from multiple databases including the Online Mendelian Inheritance in Man database (20) (OMIM),^6^ the DrugBank database (21),^7^ the GeneCards database (22)^8^ and the DisGeNet database (23).^9^ Detailed information is showed in Supplementary Table 4.

### Construction of Protein-Protein Interaction Network

PPI is short for the interaction between proteins, indicating that multiple proteins form an interaction network with non-covalent bonds. STRING 11.0 database^10^ were used to construct the PPI network of compound-CHD targets. The protein interactions were recognized as owning high confidence when the scoring value > 0.4 (24). Then we used Cytoscape 3.9.0 to merge compound-CHD target PPI network file and get the intersection of PPI network and core genes in compound-target PPI network.

### Gene Ontology and Kyoto Encyclopedia of Genes and Genomes Pathway Enrichment Analyses

The gene ontology (GO) and Kyoto Encyclopedia of Genes and Genomes (KEGG) pathway enrichment analyses were conducted using the functional annotation tool of DAVID Bioinformatics Resources.^11^ Official gene symbols were uploaded and the background was set to be homo sapiens due to the limitation of annotations. GO terms, which consists of molecular function (MF), biological process (BP) and cellular components (CC), were identified. The results of MF, BP, CC and KEGG were input into Hiplot database,^12^ which is a free and comprehensive scientific data analysis and visualization tool based on web technology and is supported by open source communities.

### Network Construction

We constructed networks using Cytoscape as follows: Firstly, the “hawthorn leaves bioactive compound target network” was established by connecting the compounds and targets of hawthorn leaves. Secondly, the “compound target signal pathway network” was constructed by connecting the targets of the ingredients and related signal pathways. Thirdly, the “PPI network” was built by connecting targets with other interactive human proteins.

### Identification of Differentially Expressed Genes Based on GSE12288

The expression profiles of GSE12288 was downloaded from the Gene Expression Omnibus (GEO) database in the NCBI portal^13^ and included for analysis. Limma package was performed to identify differentially expressed genes (DEGs) between healthy controls and coronary artery disease (CAD) samples. *P* < 0.05 were selected as thresholds to indicate a statistically significant difference. Logistic regression was performed to identify association between target genes and CAD and the model’s accuracy was evaluated using the receiver operating characteristics (ROC) curve. Statistical analyses were performed with the use of R software, version 4.0.5 (R Foundation for Statistical Computing).

### Molecular Docking Simulation

Molecular docking was performed to further demonstrate the relationship between the key compounds and major target genes. The key compounds of hawthorn leaves were downloaded from the Pubchem database (see text footnote 4) and were changed to PDB format by a software called PyMol (the PyMol Molecular Graphics System). PyMol, which is an open-source molecular visualization system, has the ability to render high-quality 3D images of small molecules and biological macromolecules, and to remove original ligand, water molecules and phosphates (25). High-quality 2D images of small molecules and biological macromolecules was used Discovery Studio (4.5 Visualizer). Next, AutoDockTools (version 1.5.6) (26) was used for preparing receptors, including adding hydrogen and setting docking parameters. The blind docking was performed with the “Grid box” set to maximum. Subsequently, we set the key compounds as ligand and used Auto-DockTools to detect their structure torsions and roots. Then we evaluated and verified the binding affinity between compounds and targets, and the reliability of predicted results from network pharmacology by Autodock Vina (27), a freely available open-source packages. The docking affinity score below –5.0 kcal/mol is recognized as the criterion which suggests there is a strong binding interaction between the compounds and their target genes (28).

### Cell Culture and Treatment

RAW264.7 cells were cultured with Dulbecco’s modified Eagle medium (DMEM, Gibco) containing 10% fetal bovine serum (Gibco) and 1% penicillin/streptomycin (Gibco) in a CO_2_ incubator (5% CO_2_ at 37°C). RAW264.7 cells were stimulated with LPS (1 μg/ml) (Sigma-Aldrich) in the presence or absence of quercetin (25, 50, 100 μM) (MCE, HY-18085), kaempferol (50, 100, 200 μM) (MCE, HY-14590), and isohamnetin (5, 10, 20 μM) (MCE, HY-N0776) for 24 h.

### Cell Viability Assay

RAW264.7 cells were inoculated in 96-well plates at 2 × 10^3^ cells/well. Following the treatments mentioned above, the Cell Counting Kit-8 solution (Promoter Biological, Wuhan, China) was added to the medium according to the operation manual, the plates were incubated for 2 h in the dark at 5% CO_2_, 37°C. The optical density (OD) was quantitated by microplate reader (Bio-Tek, Vermont, United States) at 450 nm wavelength.

### Western Blotting

IP lysate including 1:100 phenylmethanesulfonyl was used to extract RAW264.7 cells. Protein concentration was measured with a BCA kit (Boster, Wuhan, China). 10–12% gradient gels and polyvinylidene fluoride membranes were used to separate and transfer the protein samples (20–40 μg) sequentially. After blocked with 5% bovine serum albumin for 1 h, the membranes were incubated with primary antibodies against PTGS2 (1:1,000, ABClonal, Wuhan, China), EGFR (1:1,000, ABClonal, Wuhan, China), MMP2 (1:1,000, ABClonal, Wuhan, China), MMP9 (1:1,000, ABClonal, Wuhan, China), ESR1 (1:1,000, ABClonal, Wuhan, China), IL6 (1:1,000, Affinity, Jiangsu, China), IL1B (1:1,000, Proteintech, Rocky Hill, NJ, United States), TNFα (1:1,000, ABClonal, Wuhan, China) and GAPDH (1:1,000, Proteintech, Rocky Hill, NJ, United States) at 4 °C overnight. After washed with TBST, the membranes were incubated with the corresponding secondary antibodies for 1 h at room temperature. Bands were detected by Electrochemiluminescence and protein levels were analyzed with Gel Pro analysis software.

### Statistical Analysis

All statistical analyses were performed on GraphPad Prism 8.0 (GraphPad Software, United States). Significant differences were analyzed by one-way analysis of variance (ANOVA) followed by Tukey’s post-test among groups of three or more. *P* values < 0.05 were considered statistically significant.

## Results

### Identification of the Active Compounds and Target Genes in Hawthorn Leaves

**FIGURE 1:**
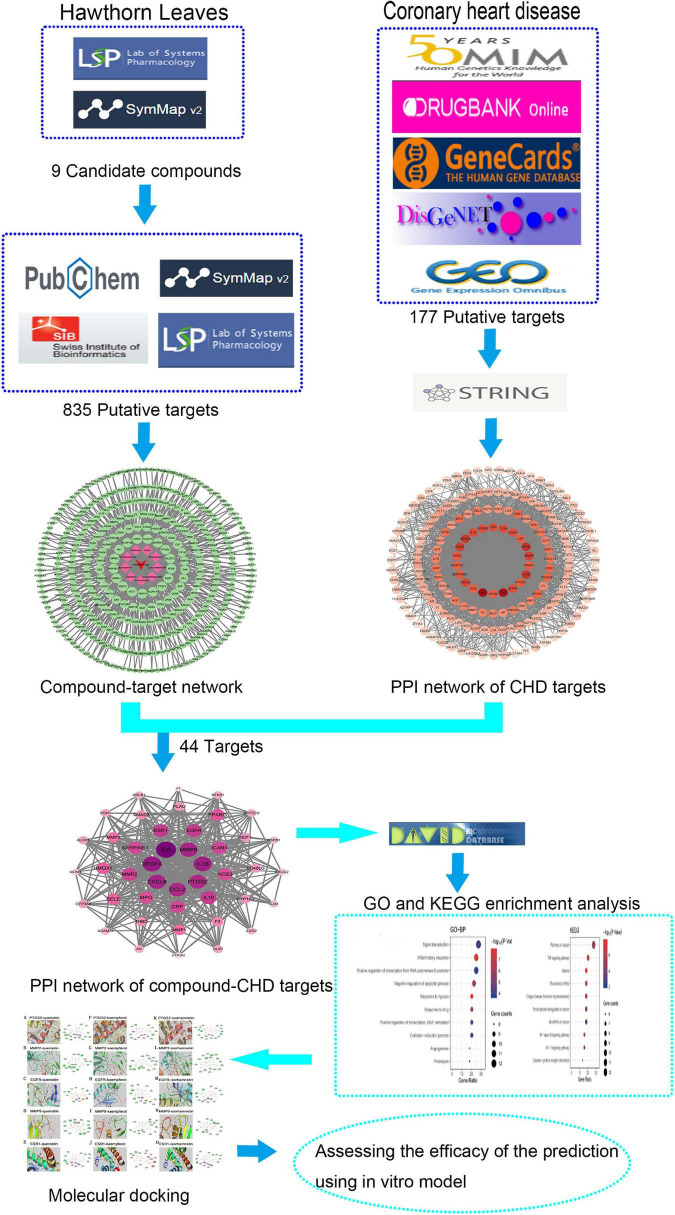
Flow diagram of investigating the mechanism of hawthorn leaves in treatment of CHD.

**FIGURE 2:**
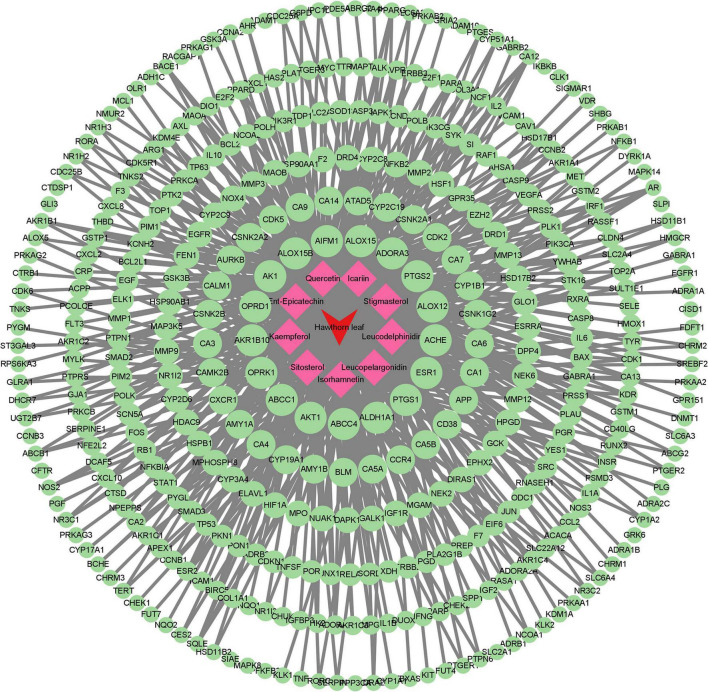
Compound–target network. Red and green circular nodes represent active compounds and corresponding targets, respectively.

The flow diagram of this study is summarized in Figure 1. With the criteria of OB ≥ 30 % and DL ≥ 0.18, 9 compounds of the hawthorn leaves were selected from TCMSP and SymMap Databases, including ent-epicatechin, quercetin, isorhamnetin, sitosterol, kaempferol, stigmasterol, leucodelphinidin, leucopelargonidin, and icariin (Table 1). Then, we identified 988 putative targets from TCMSP, Pubchem, Swiss Target Prediction and SymMap Databases. After combing all the target genes and removing the duplicated genes, we identified 835 unique target genes, the detailed information is showed in Supplementary Table 1. Subsequently, we constructed the compound-target network in order to obtain more information about the 9 compounds and their corresponding targets at the overall level (Figure 2). Adopting the average degree (93) as the threshold value, we identified top three compounds through a network topology analysis: Quercetin (degree = 310), Kaempferol (degree = 192) and Isorhamnetin (degree = 127). Moreover, 328 target genes attached to these three compounds were selected (Supplementary Table 2).

**TABLE 1:** Chemical information for the active compounds of hawthorn leaves.

Number	Compound	MW	OB(%)	DL	Composition	CAS.no	Structure
1	Ent-epicatechin	290.29	48.96	0.24	C15H14O6	35323-91-2	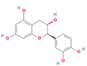
2	Quercetin	302.25	46.43	0.28	C15H10O7	117-39-5	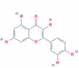
3	Isorhamnetin	316.28	49.6	0.31	C16H12O7	480-19-3	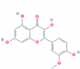
4	Sitosterol	414.79	36.91	0.75	C29H50O	83-46-5	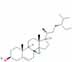
5	Kaempferol	286.25	41.88	0.24	C15H10O6	520-18-3	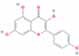
6	Stigmasterol	412.77	43.83	0.76	C29H48O	83-48-7	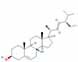
7	Leucodelphinidin	322.29	43.45	0.31	C15H14O8	55068-67-2	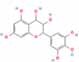
8	Leucopelargonidin	290.29	57.97	0.24	C15H14O6	520-17-2	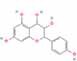
9	Icariin	676.73	41.58	0.61	C33H40O15	489-32-7	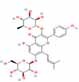

### Coronary Heart Disease Target Network

**FIGURE 3:**
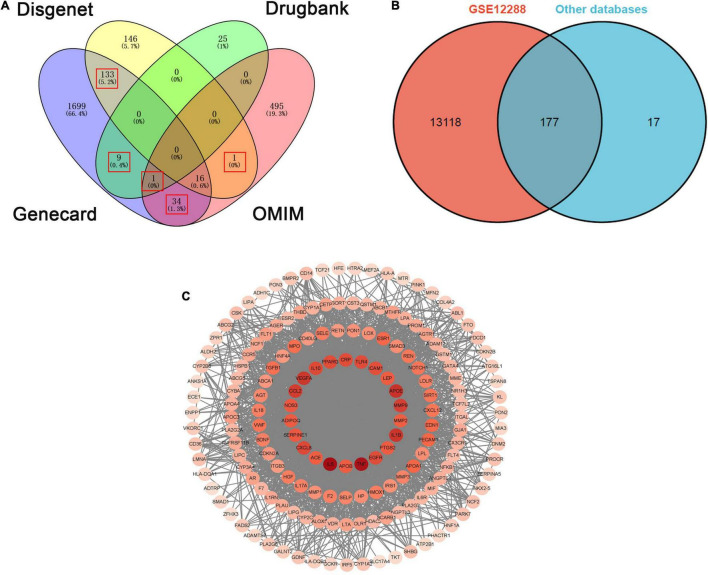
The overlapped targets of CHD from four databases and PPI network of CHD targets. **(A)** The overlapped targets of CHD from four databases (Disgenet, Dregbank, Genecard, OMIM). **(B)** The overlapped targets of CHD from GSE12288 and the other four databases. **(C)** PPI network of CHD targets.

This study got 1892, 294, 35, and 546 targets involved in CHD from Genecard, Disgenet, Drugbank, and OMIM gene database, respectively. To improve the accuracy of the selected disease targets, we selected targets that appeared simultaneously in two or more gene databases. 194 disease targets were selected in this study (Figure 3A). Moreover, the target genes of CHD were validated by using dataset of 110 patients with CAD (CADi > 23) and 112 partially matched controls without CAD (CADi = 0) that obtained in GSE12288. Finally, 177 target genes for CHD were screened out (Figure 3B). The detailed information is described in Supplementary Table 3. Moreover, the core targets were confirmed by PPI network analysis for further analysis, and the nodes with high degree values were recognized as the hub targets related to CHD. 22 targets with the highest degree values (degree ≥ two fold of the average) were IL6, TNF, APOE, IL1B, VEGFA, CCL2, MMP9, CXCL8, IL10, TLR4, CRP, PPARG, EGFR, ICAM1, SERPINE1, NOS3, LEP, ADIPOQ, PTGS2, APOB, ACE, MMP2 (Figure 3C).

### Protein-Protein Interaction Network of Compound-Coronary Heart Disease Targets

**FIGURE 4:**
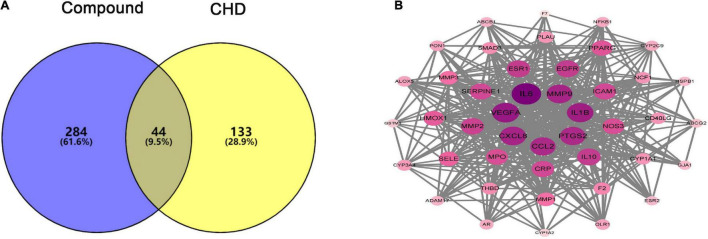
Venn diagram and PPI network of compound-CHD targets. **(A)** Venn diagram of intersecting targets of hawthorn leaves and CHD. **(B)** PPI network of compound-CHD targets.

Based on the results mentioned above, the 328 putative targets of hawthorn leaves mapped to the 177 CHD related targets to get the overlapped targets. The detailed information of CHD related targets is showed in Supplementary Table 4. Consequently, 44 targets were selected as the candidate targets responsible for CHD therapy (Figure 4A). PPI network of 44 common targets genes was constructed through STRING software (Figure 4B). 44 genes were ranked in descending order by degree (average degree = 20.32) after analyzing the topological feature of the PPI network, and the detailed information is showed in Supplementary Table 5. There were 44 nodes and 200 edges in this network. Finally, top fourteen target genes were selected out according to the topological properties of the network nodes (degree > 26). The target genes included IL6, VEGFA, IL1B, MMP9, CXCL8, CCL2, PTGS2, IL10, ESR1, EGFR, MMP2, CRP, SERPINE1 and ICAM1. These hub genes will be further used for the molecular docking study.

### Gene Ontology Analysis and Kyoto Encyclopedia of Genes and Genomes Pathway Enrichment Analysis

**FIGURE 5:**
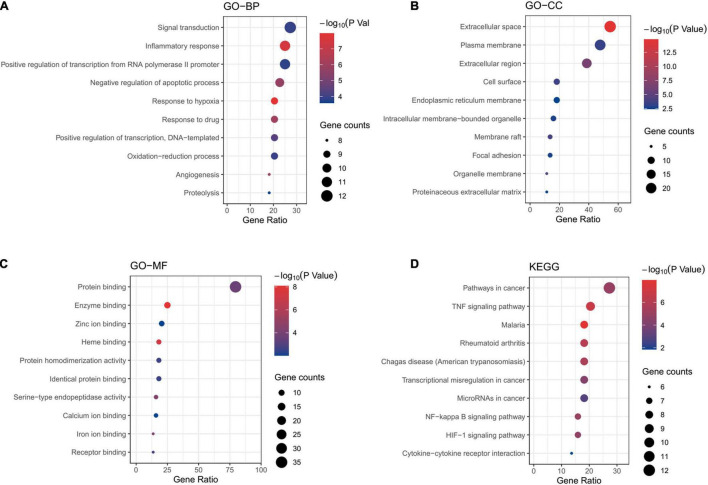
Enrichment analyses of potential targets. GO enrichment analysis. The top 10 terms of each part are shown: **(A)** GO-BP, biological processes, **(B)** GO-CC, cell component, **(C)** GO-MF, molecular function. **(D)** KEGG pathway analysis.

To clarify the mechanism of drug treatment ulteriorly, the enrichment analyses were performed. GO annotations including biological process (BP), cell composition (CC), and molecular function (MF) were analyzed. The results of enrichment included 115 BP terms, 12 CC terms and 39 MF terms. The top ten terms with a significant adjusted P-value were shown in Figures 5A–C. Main BP included the response to inflammatory response, negative regulation of apoptotic process and response to hypoxia; main CC involved extracellular space; main MF covered protein binding, enzyme binding, heme binding. 31 relevant pathways of the candidate targets were acquired through KEGG pathway enrichment (Figure 5D). The main KEGG pathways included TNF, NF-kappa B and HIF-1 signaling pathway. The detailed information is showed in Supplementary Table 6. These data suggested that the potential mechanism of action underlying CHD treatment might be the anti-inflammatory effects of hawthorn leaves.

### Molecular Docking Result Analysis

**FIGURE 6:**
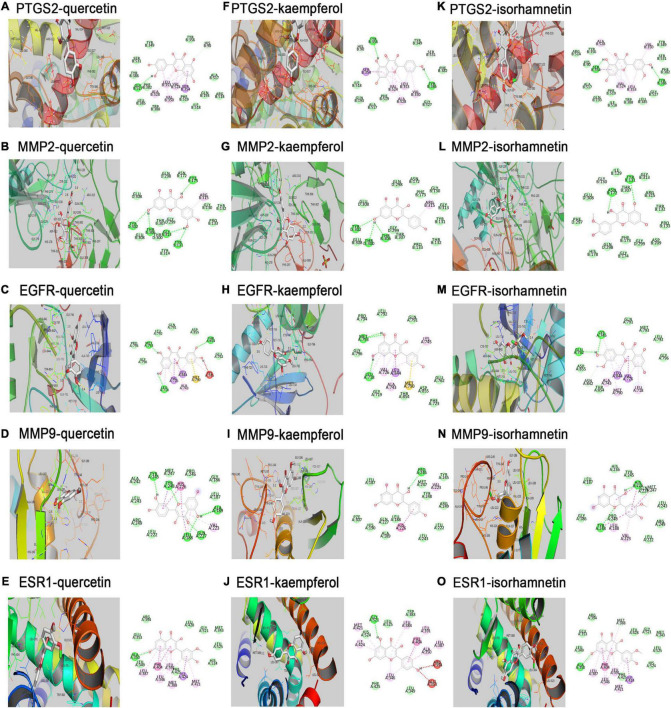
Molecular docking models of effective compounds binding to potential targets. **(A–E)** Quercetin interacting with PTGS2, MMP2, EGFR, MMP9, ESR1, respectively. **(F–J)** Kaempferol interacting with PTGS2, MMP2, EGFR, MMP9, ESR1, respectively. **(K–O)** Isorhamnetin interacting with PTGS2, MMP2, EGFR, MMP9, ESR1, respectively.

Key compounds and proteins expressed by major hub target genes were used for molecular docking. We used Autodock Vina to calculate the docking affinity score, as shown in Table 2. The Vina scores of critical compounds for treating CHD were all negative and less than -5. The results indicated that quercetin, kaempferol and isorhamnetin had good binding activities to PTGS2, EGFR, MMP2, MMP9 and ESR1. The docking conformations were shown in Figure 6. The additional docking conformations were showed in Supplementary Table 7.

**TABLE 2:** Docking scores of the active compounds of HL with their potential targets.

Compounds	Pubchem ID	Targets	PDB Code	Docking score (kcal/mol)
Quercetin	5280343	PTGS2	5ikq	–8.2
Isorhamnetin	5281654	PTGS2	5ikq	–8.5
Kaempferol	5280863	PTGS2	5ikq	–7.2
Quercetin	5280343	IL-10	2ilk	–6.5
Kaempferol	5280863	ICAM	5MZA	–7.3
Quercetin	5280343	ICAM	5MZA	–7.4
Kaempferol	5280863	EGFR	5CAP	–7.7
Isorhamnetin	5281654	EGFR	5CAP	–7.8
Quercetin	5280343	EGFR	5CAP	–8
Quercetin	5280343	MMP2	1eak	–8.3
Isorhamnetin	5281654	MMP2	1eak	–7.9
Kaempferol	5280863	MMP2	1eak	–7.9

### Quercetin, Kaempferol, Isorhamnetin Suppressed Lipopolysaccharides- Induced Inflammation and Viability in RAW264.7 Cells

**FIGURE 7:**
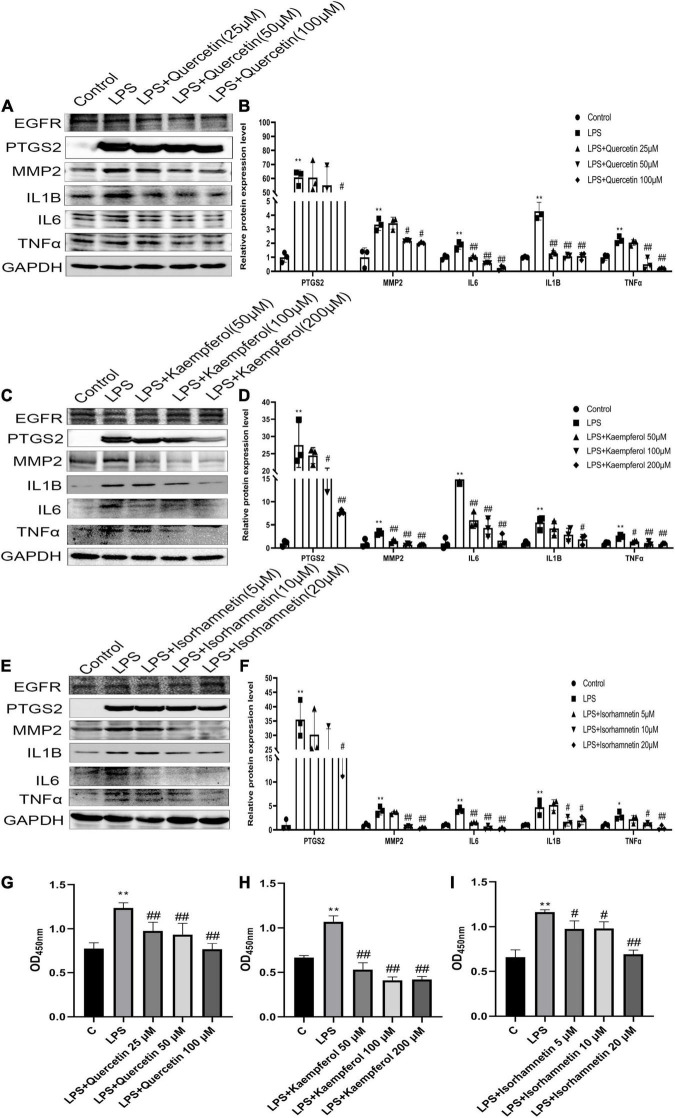
The effective compounds (quercetin, kaempferol, isorhamnetin) attenuate LPS-induced the elevation of PTGS2, MMP2 and pro- inflammation cytokines (IL1B, IL6, TNFα) of RAW264.7 cells. The protein expression of PTGS2, MMP2 and pro- inflammation cytokines (IL1B, IL6, TNFα) of RAW264.7 cells treated with quercetin **(A,B)**, kaempferol **(C,D)**, isorhamnetin **(E,F)**.The viability of RAW264.7 cells treated with quercetin **(G)**, kaempferol **(H)**, isorhamnetin **(I)**. *P < 0.05 vs. Control; **P < 0.01 vs. Control; ^#^*P* < 0.05 vs. LPS; ^##^*P* < 0.01 vs. LPS.

The PTGS2, MMP2, and MMP9 protein levels were significantly increased after exposure to LPS (1 μg/ml), while reversed by quercetin (25, 50, and 100 μM), kaempferol (50, 100, and 200 μM) and isorhamnetin (5, 10, and 20 μM) treatment, respectively, in RAW264.7 cells. However, the expression of ESR1 and EGFR did not change. Moreover, quercetin, kaempferol and isorhamnetin treatment also reduced protein level of IL6, IL1B and TNFα induced by LPS (Figures 7A–F and Supplementary Figures 1A–F). Furthermore, we also detected the effects of these three main compounds on viability of RAW264.7 cells. The data showed that all these compounds could inhibit RAW264.7 cells viability induced by LPS (1 μg/ml) (Figures 7G–I). Overall, the results indicate that the key compounds of hawthorn leaves protect against CHD through anti-inflammatory and inhibiting macrophage viability.

## Discussion

In recent years, increasing number of studies dedicated to discover new drugs and combined TCM to treat complex disease such as CHD, network pharmacology approach has been proved to play an important role in these researches. In this study, we built the following network to explore the potential targets and pathways of hawthorn leaves in the treatment of CHD: compound-target network, disease-target network, PPI network of compound-CHD targets and compound-target-pathway network. Furthermore, we verified the effects of three effective compounds on selected targets and possible mechanism *in vitro*.

After integrating information from different sources of available databases and formulating screening conditions, three main active compounds of hawthorn leaves acted on 44 different targets associated with CHD were screened out. Three compounds such as quercetin, kaempferol and isorhamnetin are highly connected with their supposed targets and can be thought as effective compounds in hawthorn leaves. As a naturally occurring polyphenol, quercetin was reported to possess antioxidant, anti-inflammatory, and antiallergic activity (29, 30). Kaempferol, which belongs to the family of flavonoids, is a secondary plant metabolite with a hydroxyl phenylchromenone structure (31). It was reported to attenuate oxidative stress and inflammatory processes in LPS-induced THP-1 cells by decreasing the mRNA expression of IL-1β, TNF, NF-κB, and IL6 level (32). Isorhamnetin, which is extracted from the fruits of hippophae rhamnoides L and the leaves of Ginkgo biloba L, is an important natural flavonoid (33), and has been shown to possess both anti-microbial and anti-inflammatory properties (34). Therefore, these suggest hawthorn leaves may have beneficial effects on CHD which is characterized by immune inflammatory response.

Actually, consistent with prior researches, the network of CHD targets in our study did reveal the underlying pathogenesis of CHD. Abundant data confirmed that inflammatory mechanisms play a vital role in the occurrence and development of atherosclerosis and CHD (35). Early atherogenesis is characterized by recruitment of leukocyte and expression of pro-inflammatory cytokines, the inflammation also promotes plaque rupture and thrombogenesis, which lead to myocardial infarctions and most strokes (36). Exploring the role of inflammation in atherosclerosis also provides a new perspective on the underlying mechanisms behind the clinical benefits of lipid-lowering therapy. All of these researches suggest that identifying triggers of inflammation and elucidating the details of inflammatory pathways may provide new therapeutic targets for treating CHD (37).

To better elucidate the mechanism of hawthorn leaves in the treatment of CHD, the 44 targets were screened out in the compound-CHD targets. Coincidentally, 14 targets, namely IL6, VEGFA, IL1B, MMP9, CXCL8, CCL2, PTGS2, IL10, ESR1, EGFR, MMP2, CRP, SERPINE1, and ICAM1, have been identified as key pathogenic genes in the PPI network of CHD target proteins. These results reveal that hawthorn leaves might protect against CHD through a multi-targets synergistic way.

As shown by the compound-target gene-pathway network, quercetin, kaempferol and isorhamnetin interacted with lots of targets, indicating their complex and comprehensive roles in the treatment of CHD. To further understand the binding ability between compounds and targets, molecular docking simulation was performed between 14 targets genes and three active compounds to find out major targets. Absolute value of the docking affinity are positively correlated with binding force between the compounds and the active site of targets. The data showed that some of the compound- target pairs had good docking affinity, such as, PTGS2, MMP2, EGFR, MMP9, and ESR1 (Figure 6), which means that the protective effects of hawthorn leaves in CHD may be achieved to some extent by regulating these genes above. PTGS2, a key enzyme in prostaglandin biosynthesis, is involved in prostaglandin biosynthesis and plays a significant role in inflammatory response (38, 39). Prior study has noted that PTGS2 may be potential therapeutic target for CHD (40), but there is no report confirming it. MMP2 and MMP9 are a kind of proteases that have been reported to mediate plaque rupture and inflammation in atherosclerosis (41–43). Previous study showed that selective EGFR deletion in myeloid cells limited their ability to produce IL-6, TNF-α, and to uptake lipids, thereby reducing the development of atherosclerosis (44). ESR1 was also reported to be related to the risk of CHD, one study showed that ESR1 genetic variants were associated with the increased risk of CHD among Finnish men (45). Our results demonstrated that the increased PTGS2, MMP2, and MMP9 protein levels induced by LPS could be reversed by quercetin, kaempferol and isorhamnetin, respectively, in RAW264.7 cells. However, the expression of EGFR and ESR1 did not change. These data suggested that PTGS2, MMP2 and MMP9 might be involved in the protection of hawthorn leaves against CHD. In addition, we added the ROC analysis for PTGS2 and MMP2 genes using logistic regression models based on GSE12288 datasets (Supplementary Figure 1G–J). However, there is no difference in these data, and we think there are several reasons: First, gene expression in circulating leukocytes was measured to identify patients with CAD or the controls in GSE12288 database. Therefore, differential genes do not necessarily play a role in CHD. Similarly, the non-differential genes in the database do not mean that they will not play an important role in the development of CHD. Second, it cannot rule out the possibility that these two genes are expressed differently in the myocardium of CHD patients and normal controls.

The potential therapeutic mechanism was also validated by GO functional annotation and pathway enrichment analysis. First, main BP in GO pathway enrichment analysis of common-targets included signal transduction and the response to inflammatory response, which means most key targets participate in these two biological process; Second, main CC of common-targets included extracellular space, plasma membrane, which means those targets that we may be interested in mostly locate at extracellular space and plasma membrane; Third, main MF of these targets is protein banding, which means the function of these targets is mainly protein binding. Besides, the results of KEGG pathway enrichment analysis showed that effects of hawthorn leaves on CHD may be due to its ability to target varied pathways simultaneously, such as pathways in cancer, TNF signaling pathway, malaria, and so on (Figure 5D). As for TNF signaling pathway, a classic pathway involved in inflammatory response, IL1β, IL6 and TNFα are key members of it. IL6 was verified to implicate in the pathogenesis of CHD, possibly due to its association with inflammation, insulin resistance, and plasma ceramides (46). It has been reported that reducing the production of IL6 mediated by ROS/p38//NF-κB signaling pathway can attenuate atherosclerosis (47). Inhibiting IL-6/STAT3 signaling pathway can protect against high-fat-induced atherosclerosis in ApoE (-/-) mice (47). IL-1β could activate NF-κB signaling pathway (48), leading to inflammation cascades through increasing the production of pro-inflammatory cytokines (TNF-α and IL-6) (49). Our research showed that quercetin, kaempferol and isorhamnetin reduced protein expression of IL-1β, IL6, TNFα induced by LPS in RAW264.7 cells. Additionally, the macrophages activation was suppressed by these three compounds mentioned above. These data further manifest that the effective compounds of hawthorn leaves could inhibit the production of pro-inflammatory cytokines stimulated by LPS in macrophage, confirming that hawthorn leaves may play a therapeutic role in CHD by inhibiting inflammatory pathways. However, about the effect of LPS on the viability of macrophage, there are some different views. Some studies suggested that LPS had no significant effect on the viability of macrophages (50–54), some literatures mentioned that LPS could reduce the viability of macrophages (55, 56), while other studies showed that LPS could increase the viability of macrophages (57, 58). Consistent with the latter, our results showed that LPS could increase the viability of macrophages. Through reviewing the literature, we found that these different results may be related to the differences in the time point or dose of LPS intervention. More confusingly, studies with exactly the same LPS dose and time of intervention had inconsistent results. For example, when RAW264.7 were interfered with 1ug/mL LPS for 24 h in three studies, the first study showed that LPS decreased the viability of cells (55), the second study showed that LPS increased the viability of cells (58), and the third study showed that LPS didn’t affect the viability of cells (53). One possible explanation is that the effectiveness of LPS from different sources may be different. We have to admit that we are puzzled by this complex situation, and further researches may be needed to clarify the causes of these different effects.

The results of our study are consistent with that of previous similar study which explored the effects of hawthorn leaves on different disease models. Fu et al. reported that hawthorn leaves flavonoids could decrease the degree and scope of myocardial ischemia by inhibiting inflammation in anesthetized dogs with acute myocardial ischemia/reperfusion (59). Min et al. reported hawthorn leaves flavonoids could protect against diabetic cardiomyopathy in rats through reducing oxidative stress and inflammation (60). The result of another study also showed that vitexin which is the active ingredient of hawthorn leaves extract could decrease DOX−induced cardio-toxicity by inhibiting inflammation, oxidative stress and apoptosis (61). These results further prove that hawthorn leaves really have a significant inhibitory effect on inflammation. Different from these studies, our study adopted the method of network pharmacology analysis to screen the effective components and action targets of hawthorn leaves in the treatment of CHD, and verified that the anti-inflammatory effect may be the key mechanism of its effect through *in vitro* experiments. This study further proves that network pharmacology is an effective method to screen the active components and action targets of TCM, and provides methodological inspiration for other studies about TCM in the future.

In fact, besides anti-inflammatory action, non-anti-inflammatory effects have been considered by several studies to be involved in the beneficial effects of hawthorn leaves. Previous studies have shown that hawthorn leaves could improve non-alcoholic fatty liver disease and diabetic cardiomyopathy by inhibiting oxidative stress (60, 62). Another study suggested that hawthorn leaves attenuated hepatic steatosis in high-fat fed rats by enhancing the Adiponectin/AMPK pathway (63). It is worth noting that one study indicated that hawthorn leaves may also have a blood thinning effect in addition to antioxidant stress. Therefore, for patients with heart diseases who require routine antiplatelet and anticoagulant drugs, they should be aware of the risk of bleeding when taking medicine containing hawthorn leaves (64).

However, there are still some limitations in this research. Firstly, as a kind of complex disease, the underlying mechanisms behind the occurrence and progression of CHD are complicated, the mechanism of action of hawthorn leaves in treatment of CHD needs more comprehensive *in vitro* and *in vivo* researches. Secondly, although three targets (PTGS2, MMP9 and MMP2) and three inflammatory factors (IL-1β, IL6, TNFα) were verified to be involved in mechanism of action, the potential upstream or downstream relationships between them still need to be further explored. Thirdly, we have only studied the anti-inflammatory effects and possible mechanisms of hawthorn leaves in macrophages, but lack of studies in endothelial cells and smooth muscle cells. Fourth, the results of enrichment analysis suggested that hawthorn leaves may also have non-anti-inflammatory effects, and it may be biased to focus only on its anti-inflammatory effects. Therefore, in order to better understand its potential mechanism of action, the non-anti-inflammatory effects of hawthorn leaves should be further studied in future.

## Conclusion

**FIGURE 8:**
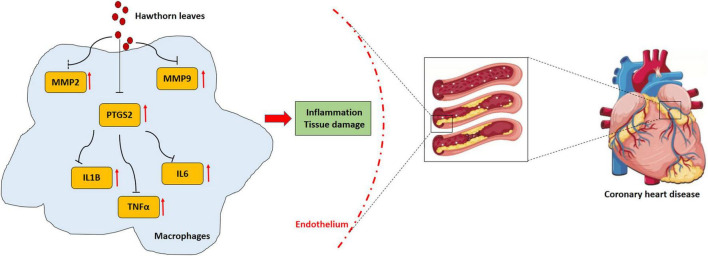
The working model shows the mechanism of hawthorn leaves against CHD.

In conclusion, the effective components and key targets of hawthorn leaves for CHD treatment were explored using network pharmacology approach. Quercetin, kaempferol and isorhamnetin were proved to be the main effective compounds in treatment of CHD, possibly by suppressing expression of PTGS2, MMP2, MMP9, inflammatory cytokines and activation of macrophages (Figure 8). The present study provides a more comprehensive understanding of hawthorn leaves on active components and targets, which is conducive to further optimization of the drug in the future.

## Data Availability Statement

The original contributions presented in the study are included in the article/Supplementary Material, further inquiries can be directed to the corresponding author/s.

## Author Contributions

QF designed the experiments, wrote the manuscript, and conducted the pharmacology information analyses. JW helped to conduct the pharmacology information analyses. SL and MH participated in visualizing the data. HRW helped with the data analyses. JD completed the *in vitro* experiments and helped to write and review the manuscript. YW organized the study. All authors read and approved the final manuscript.

## Conflict of Interest

The authors declare that the research was conducted in the absence of any commercial or financial relationships that could be construed as a potential conflict of interest.

## Publisher’s Note

All claims expressed in this article are solely those of the authors and do not necessarily represent those of their affiliated organizations, or those of the publisher, the editors and the reviewers. Any product that may be evaluated in this article, or claim that may be made by its manufacturer, is not guaranteed or endorsed by the publisher.

## Footnotes

1
https://old.tcmsp-e.com/tcmsp.php

2
http://www.symmap.org/

3
http://pubchem.ncbi.nlm.nih.gov

4
http://www.swisstargetprediction.ch/

5
http://www.uniprot.org/

6
http://omim.org/

7
https://www.drugbank.ca/

8
https://www.genecards.org/

9
https://www.disgenet.org/home/

10
https://string-db.org/

11
https://david.ncifcrf.gov/search.jsp

12
https://hiplot.org

13
ncbi.nlm.nih.gov

## Supplementary Material

Supplementary Figure 1 The effective compounds (quercetin, kaempferol, isorhamnetin) attenuate LPS-induced the elevation of MMP9 in RAW264.7 cells. The protein expression of MMP9 and ESR1 of RAW264.7 cells treated with quercetin **(A,B)**, kaempferol **(C,D)**, isorhamnetin **(E,F)**. Analysis of gene differential expression and ROC analyses for PTGS2 and MMP2 genes based on the data from GSE12288 **(G–J)**. **P* < 0.05 vs. Control; ^**^*P* < 0.01 vs. Control; ^#^*P* < 0.05 vs. LPS; ^##^*P* < 0.01 vs. LPS.

## Abbreviations

CHD: coronary heart disease
LPS: lipopolysaccharides
TCM: Traditional Chinese Medicine
TCMSP: Traditional Chinese Medicine Systems Pharmacology
ADME: absorption distribution, metabolism and excretion
OB: oral bioavailability
DL: drug-likeness
OMIM: Online Mendelian Inheritance in Man
PPI: Protein-protein interaction
GO: Gene Ontology
KEGG: Kyoto Encyclopedia of Genes and Genomes
MF: molecular function
BP: biological process
CC: cellular components
CAD: Coronary artery disease
PyMol: PyMol Molecular
DMEM: Dulbecco’s modified Eagle medium
OD: optical density
ANOVA: one-way analysis of variance.
