# Supplementary figures and images for: Exploring the Mechanism of Hawthorn Leaves Against Coronary Heart Disease Using Network Pharmacology and Molecular Docking
Source: Front Cardiovasc Med. 2022 Jun 16;9:804801. doi: 10.3389/fcvm.2022.804801 (PMC9243333; doi:10.3389/fcvm.2022.804801)

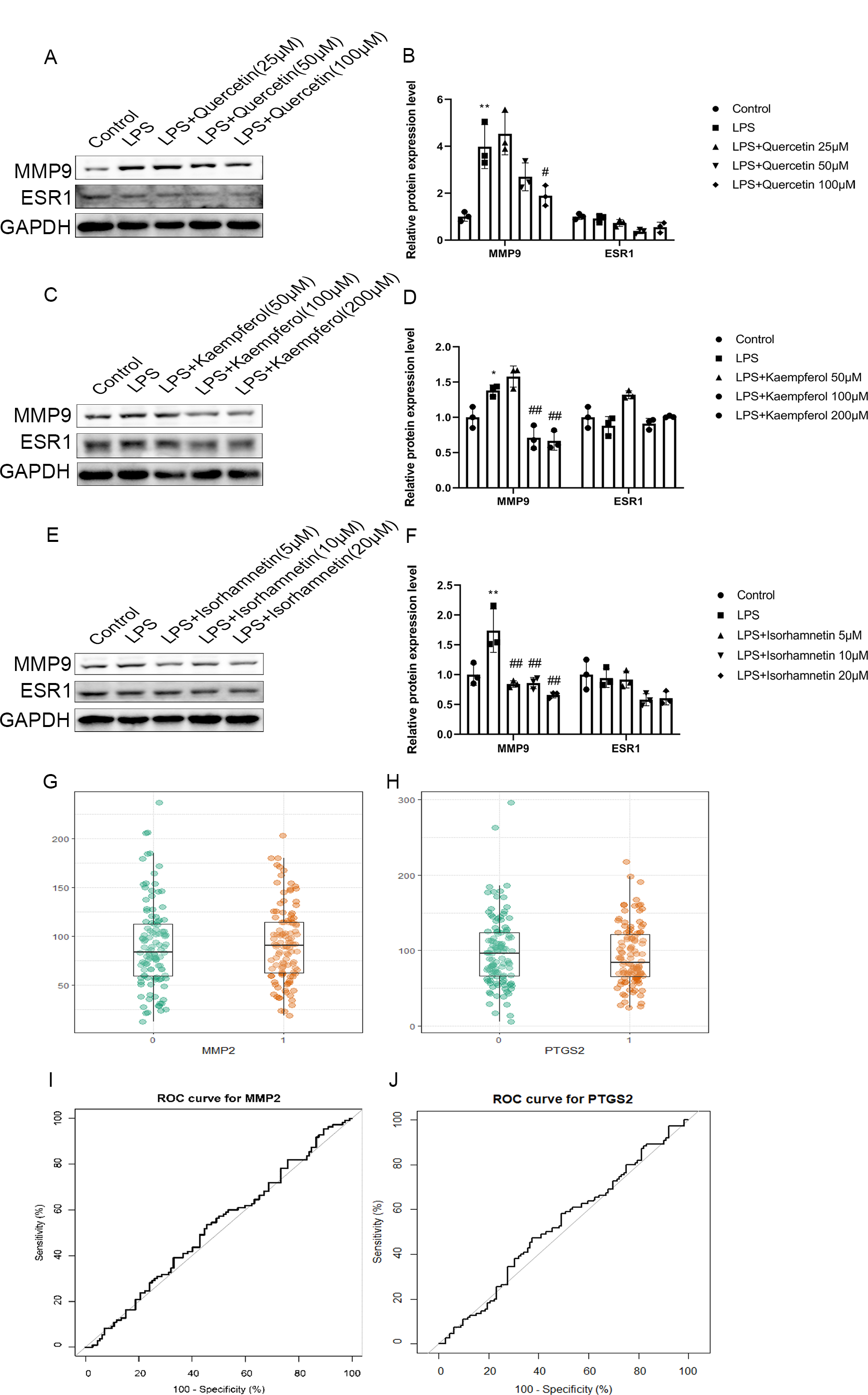

Supplement: Supplementary Figure 1 — The effective compounds (quercetin, kaempferol, isorhamnetin) attenuate LPS-induced the elevation of MMP9 in RAW264.7 cells. The protein expression of MMP9 and ESR1 of RAW264.7 cells treated with quercetin (A,B), kaempferol (C,D), isorhamnetin (E,F). Analysis of gene differential expression and ROC analyses for PTGS2 and MMP2 genes based on the data from GSE12288 (G–J). *P < 0.05 vs. Control; **P < 0.01 vs. Control; #P < 0.05 vs. LPS; ##P < 0.01 vs. LPS. [file Image_1.TIF]
